# Tissue-Engineered Small Diameter Arterial Vascular Grafts from Cell-Free Nanofiber PCL/Chitosan Scaffolds in a Sheep Model

**DOI:** 10.1371/journal.pone.0158555

**Published:** 2016-07-28

**Authors:** Takuma Fukunishi, Cameron A. Best, Tadahisa Sugiura, Toshihiro Shoji, Tai Yi, Brooks Udelsman, Devan Ohst, Chin Siang Ong, Huaitao Zhang, Toshiharu Shinoka, Christopher K. Breuer, Jed Johnson, Narutoshi Hibino

**Affiliations:** 1 Department of Cardiac Surgery, Johns Hopkins University, Baltimore, MD, United States of America; 2 Tissue Engineering and Center for Cardiovascular and Pulmonary Research, Nationwide Children’s Hospital, Columbus, OH, United States of America; 3 Yale University School of Medicine, New Haven, CT, United States of America; 4 Nanofiber Solutions Inc, Columbus, OH, United States of America; Monash University, AUSTRALIA

## Abstract

Tissue engineered vascular grafts (TEVGs) have the potential to overcome the issues faced by existing small diameter prosthetic grafts by providing a biodegradable scaffold where the patient’s own cells can engraft and form functional neotissue. However, applying classical approaches to create arterial TEVGs using slow degrading materials with supraphysiological mechanical properties, typically results in limited host cell infiltration, poor remodeling, stenosis, and calcification. The purpose of this study is to evaluate the feasibility of novel small diameter arterial TEVGs created using fast degrading material. A 1.0mm and 5.0mm diameter TEVGs were fabricated with electrospun polycaprolactone (PCL) and chitosan (CS) blend nanofibers. The 1.0mm TEVGs were implanted in mice (n = 3) as an unseeded infrarenal abdominal aorta interposition conduit., The 5.0mm TEVGs were implanted in sheep (n = 6) as an unseeded carotid artery (CA) interposition conduit. Mice were followed with ultrasound and sacrificed at 6 months. All 1.0mm TEVGs remained patent without evidence of thrombosis or aneurysm formation. Based on small animal outcomes, sheep were followed with ultrasound and sacrificed at 6 months for histological and mechanical analysis. There was no aneurysm formation or calcification in the TEVGs. 4 out of 6 grafts (67%) were patent. After 6 months *in vivo*, 9.1 ± 5.4% remained of the original scaffold. Histological analysis of patent grafts demonstrated deposition of extracellular matrix constituents including elastin and collagen production, as well as endothelialization and organized contractile smooth muscle cells, similar to that of native CA. The mechanical properties of TEVGs were comparable to native CA. There was a significant positive correlation between TEVG wall thickness and CD68^+^ macrophage infiltration into the scaffold (R^2^ = 0.95, *p* = 0.001). The fast degradation of CS in our novel TEVG promoted excellent cellular infiltration and neotissue formation without calcification or aneurysm. Modulating host macrophage infiltration into the scaffold is a key to reducing excessive neotissue formation and stenosis.

## Introduction

Coronary artery disease (CAD) and peripheral vascular disease (PVD) are leading causes of death and impaired quality of life. Vascular graft transplantations, such as coronary artery bypass grafts (CABG) and distal limb bypass [1], are common operations performed in approximately 600,000 patients annually in the USA [2]. The autologous great saphenous vein and internal mammary artery are small diameter grafts (<6mm) widely accepted as the current gold standard for CABG surgery. These autografts have several disadvantages in patients such as inherent size mismatches, pre-existing vascular disease (such as atherosclerosis), and supply shortage due to previous procedures. Expanded polytetrafluoroethylene (ePTFE, Gore-Tex^®^) is the most commonly used synthetic graft material for distal limb bypass, and has been clinically successful in situations where the graft diameter is larger than 6mm (high flow, low resistance circulation) [3–5]. However, its efficacy is limited in small diameter vascular grafts (<6mm) due to thrombosis (platelet adhesion and aggregation), progressive stenosis (neointimal hyperplasia and over-proliferation of smooth muscle cells), calcium deposition, host rejection, increased risk of infection and the need for anticoagulation therapy [6–12].

Small diameter tissue engineered vascular grafts (TEVGs) have been developed to withstand the high pressures of the arterial circulation. In order to create functional compliance, the formation of well-organized vascular neotissue through the vascular remodeling process is paramount.[13–16]. Therefore, an ideal graft will undergo rapid vascular remodeling by facilitating cellular infiltration and scaffold degradation. Recently, three-dimensional porous nanofiber scaffolds fabricated by electrospinning have been widely investigated as arterial TEVGs [17, 18]. Previous studies demonstrated that slow degrading polycaprolactone (PCL) or polylactic acid (PLA) can provide adequate mechanical properties as small diameter TEVGs when applied to the abdominal aorta in the mouse and rat models. However, slow degrading PCL grafts had limited cell infiltration, poor neotissue formation, and localized calcification in the long-term [13, 19, 20].

Recent studies have investigated materials fabricated by combining both synthetic polymers and natural proteins, such as collagen, elastin, gelatin, chitosan (CS). These hybrid scaffold materials have promising potential for the next generation of small diameter arterial TEVGs [21–24] because of their fast degradation and hydrophilic characteristics that promote better cell infiltration, proliferation and neotissue formation. CS and synthetic polymer combinations have been successfully used as tissue engineering scaffolds for bone, cartilage and skin, but little is known about their potential for tissue engineered vascular grafts, especially in large animal models [17, 18, 25].

The purpose of this study is to investigate the feasibility of unseeded (cell-free) small diameter hybrid TEVGs (5mm) fabricated by electrospinning a blend of synthetic PCL and natural CS in small and large animal models of high pressure circulation over a 6 month time course.

## Materials and Methods

### Study design

We evaluated the efficacy of a novel cell-free nanofiber PCL/chitosan TEVGs as arterial conduits implanted in mice at the end of 6 months. The TEVGs were implanted into mice (n = 3) as an infrarenal abdominal aorta (IAA) interposition grafts and all demonstrated patency without graft narrowing or rupture (100%). We subsequently implanted the TEVGs in sheep (n = 6) as carotid artery (CA) interposition grafts. The TEVGs were explanted at 6 months post-implantation for evaluation of neotissue formation, biocompatibility and mechanical properties. There were no instances of data exclusion in this study and outliers were also included in the data analysis.

### Scaffold fabrication

Polycaprolactone (PCL) (Mn 70,000–90,000) and chitosan (CS) (Medium molecular weight) were purchased from Sigma Aldrich Co, LLC (Missouri, USA). Hexafluoroisopropanol (HFIP) and acetic acid (AA) (99.7%) were purchased from Oakwood Chemicals and Sigma Aldrich, respectively. All polymers and solvents were used without further modification.

Chitosan (CS) was dissolved in a 9:1 w/w solution of hexafluoroisopropanol (HFIP) and acetic acid to produce a 0.15 wt% CS solution and was stirred via a magnetic stir bar for 3 hours at 50°C. Polycaprolactone (PCL) was then added to the CS solution in a 20:1 w/w ratio of PCL and CS and stirred via a magnetic stir bar for 21 hours at 50°C. The PCL/CS solution was placed in a 20cc luer-lok syringe and dispensed at a flow rate of 5ml/h through a 20 gauge blunt needle tip. A 1.0mm diameter mandrel was used for the mouse grafts while a 5.0mm diameter mandrel was used for the ovine grafts. The mandrel was positioned 20cm from the needle tip and was rotated at 100 RPM. A +25kV charge was applied to the needle tip and a -4kV charge was applied to the mandrel. The electrospun nanofibers were deposited to create a 100 ± 10μm wall thickness tube for the murine grafts and 400 ± 40μm wall thickness tube for the ovine grafts. The PCL/CS tubes were cut into 0.3cm lengths for the murine grafts and 1.5cm lengths for the ovine grafts. Finally, all the PCL/CS TEVGs were terminally sterilized with 50kGy of gamma irradiation.

### Mechanical testing

Compliance and burst pressure data were acquired as previously described [26]. In brief, data was acquired using an MTS Systems Corporation (Minnesota, USA) load frame fitted with a 50 lb load cell with a force resolution of 10^−4^ pounds and a linear displacement resolution of 10^−8^ inches. Compliance testing was performed using a displacement velocity of 1.5 mm per minute and acquisition rate of 4 data points per second utilizing Laplace’s Law [27, 28] to correlate linear force and displacement to compliance. Burst pressure testing was performed using a displacement velocity of 50 mm per minute and acquisition rate of 4 data points per second utilizing Laplace’s Law [28, 29] to correlate linear force and displacement to burst pressure.

Samples were placed around two parallel L-shaped steel rods, one rod was attached to the base of the universal testing machine and the other to the load cell. The samples were strained perpendicular to the length of the sample. Compliance was calculated using systolic and diastolic pressures of 120 mmHg and 80 mmHg, respectively. Burst pressure was calculated using the maximum force before failure.

### Degradation rate *in vitro*

ASTM D638 Type V tensile dogbones were cut from sheets of the PCL/CS nanofiber and placed in Ringers solution and incubated at 37°C to mimic *in vivo* hydrolytic degradation conditions. Mass and mechanical properties were measured at 1, 2, 3 and 4 weeks to determine the degradation profile of this nanofiber composite material. Mass loss was determined by drying each sample under vacuum for 72 hours before weighing. Tensile testing was performed on an MTS Systems Corporation (Minnesota, USA) load frame with a 10N load cell and an elongation rate of 15mm/min. 5 samples were used for each test at each time point.

### Graft implantation

All mice procedures received prior approval from the institutional animal care and use committee at Yale University. Nanofiber-PCL/CS TEVGs were implanted as interpositional grafts in the infrarenal abdominal aorta (IAA) in 3 female CB-17 severe combined immunodeficient-beige (SCID/bg) mice (n = 3). Implantation was accomplished using a sterile microsurgical technique described previously[30]. Briefly, anesthetized mice were placed in the supine position and opened with an abdominal midline incision. A portion of the infrarenal abdominal aorta was exposed, cross-clamped, and excised. Three-millimeter length scaffolds were then inserted as interposition grafts using a running 10–0 nylon suture for the end-to-end proximal and distal anastomoses. TEVG patency, wall thickness, and lumen diameter were assessed *in vivo* using high frequency ultrasound system (Vevo 770, Visualsonics, Toronto, Canada) with a RMV-704 transducer until the 6 months end point.

The animal care and use committee at the Q-Test Laboratories (Columbus, Ohio) approved the care, use, and monitoring of animals for sheep experiments. Nanofiber-PCL/CS TEVGs were implanted as right carotid artery interposition grafts in sheep (n = 6, body weight: 23.9 ± 5.0 kg). All sheep were anesthetized with 1.5% isofluorane during surgery. The carotid artery was exposed and heparin (100 IU/kg) was administered intravenously. The TEVG was implanted as a carotid artery interposition graft using standard running 7–0 prolene suture (Ethicon Inc.). Hemostasis was obtained, and the muscle, subcutaneous tissue, and dermal incision layers were closed. Antibiotic treatment (cefazolin) was administered intra-operatively and 7 days post-operatively. All sheep were maintained on a daily oral medication of aspirin (325 mg/day) until the 6 months end point. Color Doppler ultrasound was performed at 1, 3 and 6 months to determine graft patency and lumen diameter. Animals were euthanized using pentobarbital sodium, and grafts were explanted at 6 months after surgery.

### Histology and immunohistochemistry

Explanted TEVG samples were fixed in 10% formalin for 24 hours at 4°C, then embedded in paraffin. For standard histology, tissue sections were stained with hematoxylin and eosin (H&E), Masson’s trichrome, picrosirius red, Hart’s, and von Kossa. For immunohistochemistry, tissue sections were deparaffinized, rehydrated, and blocked for endogenous peroxidase activity and nonspecific staining. The primary antibodies used included: von Willebrand Factor (vWF, Dako, 1:2000), α-smooth muscle actin (SMA, Dako, 1:500), calponin (Abcam, 1:500), myosin heavy chain (MHC, Abcam, 1:500), CD68 (Abcam, 1:200). Antibody binding was detected using biotinylated secondary antibodies, followed by incubation with streptavidinated HRP. Color development was performed by chromogenic reaction with 3,3-diaminobenzidine (Vector). Nuclei were counterstained with hematoxylin (Gill’s formula, Vector).

### Histological and quantitative analysis

The lumen diameter, wall thickness, remaining scaffold area, and collagen quantitative analyses were measured from H&E and picrosirius red staining using Image J software (Image Processing and Analysis in Java; National Institutes of Health, Bethesda, MD, USA). Macrophages, identified by positive CD68 expression, were quantified for each explanted scaffold. Four sections of each individual sample were counted at 40x (high powered field; HPF) and averaged for a total of six samples.

### Statistical analysis

For all experiments, data are represented as mean ± SEM, and statistically significant differences between groups were determined using Student’s t-test and Pearson correlation test. A paired t-test was performed comparing native tissue to tissue-engineered samples. A *p*-value less than 0.05 was considered statistically significant. Statistical analysis was performed using Graphpad Prism (GraphPad Software, Inc., version 6, CA, USA).

## Results

### Scaffold characterization

**Fig 1:**
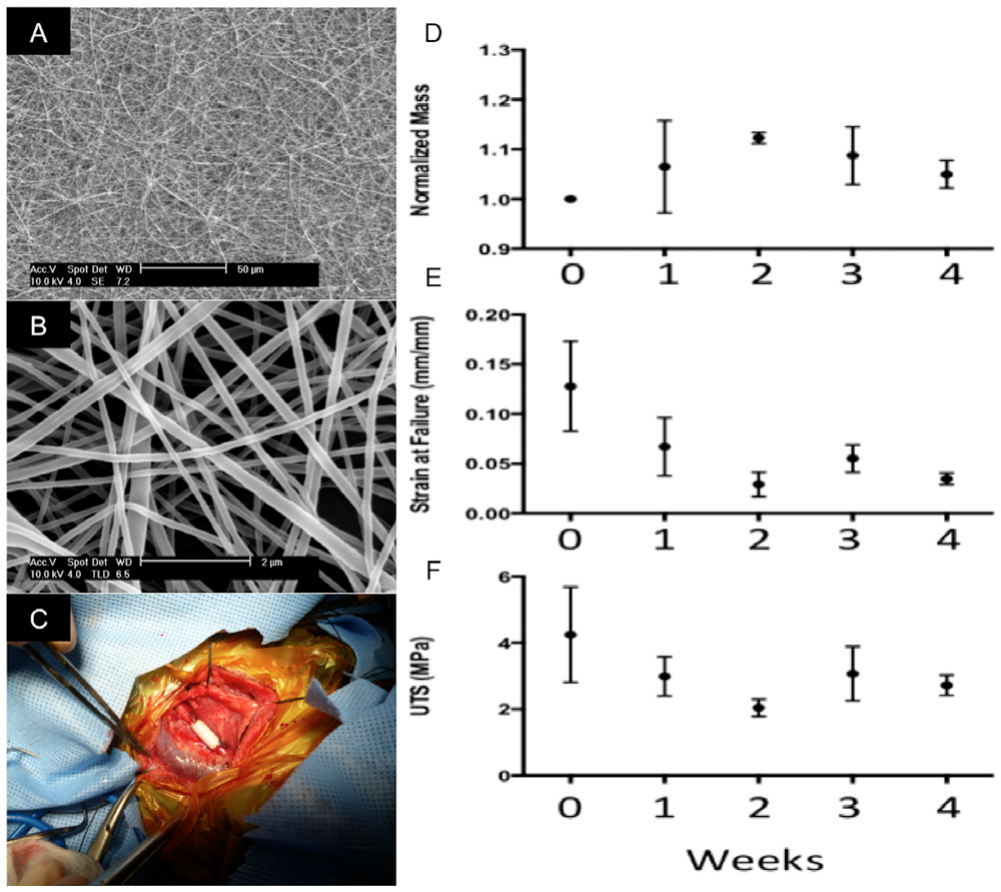
Characterization of Scaffolds. Representative scanning electron microscopy of PCL/CS TEVGs prior to implantation at low powered (A) and high powered fields (B). Perioperative image of freshly implanted scaffold (C). *In vitro* degradation of PCL/CS measured by change in mass (D), change in strain at failure (E) and change in UTS (F) (n = 5).

Scanning electron microscopy revealed very fine uniform fibers with a diameter of 150nm ± 62nm (Fig 1A and 1B). The slow mandrel rotation speed allowed the scaffold to have no preferential alignment of fibers and isotropic properties. Additionally, the PCL and chitosan were blended within each fiber, rather than a scaffold comprised of monolithic PCL fibers and monolithic chitosan fibers (Fig 1C). *In vitro* characterization of PCL/CS scaffold degradation through 4 weeks demonstrated an approximately 50% loss in ultimate tensile strength (UTS) by 2 weeks with a similar decrease in strain at failure that was followed by a plateauing out to 4 weeks (Fig 1E and 1F). In contrast, mass remained constant throughout the experimental time course (Fig 1D).

### Nanofiber PCL/chitosan TEVGs in a mouse model

**Fig 2:**
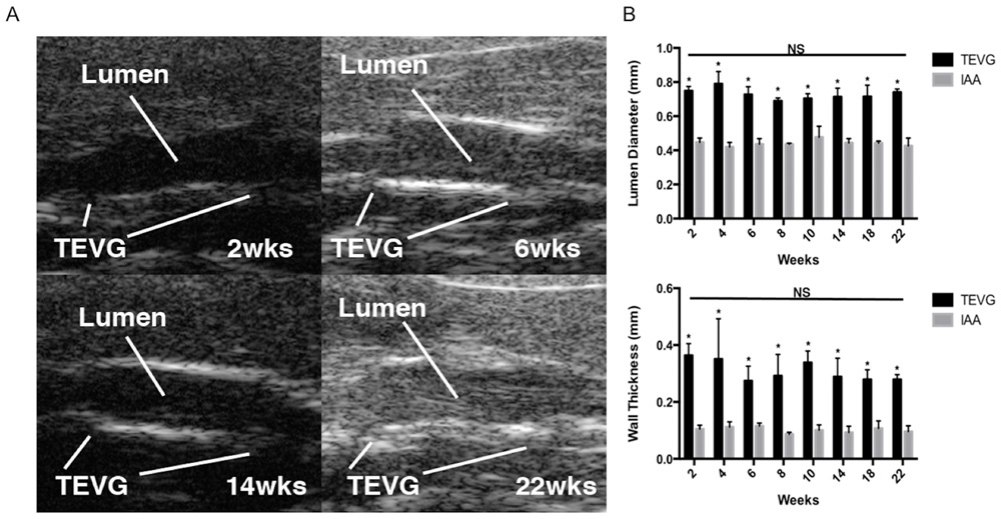
Preliminary study results. Representative Doppler ultrasound images of TEVGs at various time points showing graft patency *in vivo* (A). TEVG lumen diameter and wall thickness as measured by Doppler ultrasound (n = 3). Data in graphs are expressed as mean ± SD. *P <0.05. There was a statistically significant difference in both luminal diameter and wall thickness between TEVG and IAA at each time point. However, no statistically significant difference (NS) was found between all time points for either TEVG or native IAA (B).

Cell-free nanofiber PCL/CS scaffolds were successfully implanted as interpostional IAA grafts and all mice survived until the 6 months end point without complications. All TEVGs remained patent without evidence of thrombosis or aneurysm formation despite initial graft oversizing of 50% (Fig 2A). With regards to lumen diameter and wall thickness, Doppler ultrasound revealed no evidence of graft failure, and no statistically significant differences were found between time points for both the TEVG or native IAA (Fig 2B).

### Nanofiber PCL/chitosan TEVGs in a sheep model

#### Mechanical properties

**Fig 3:**
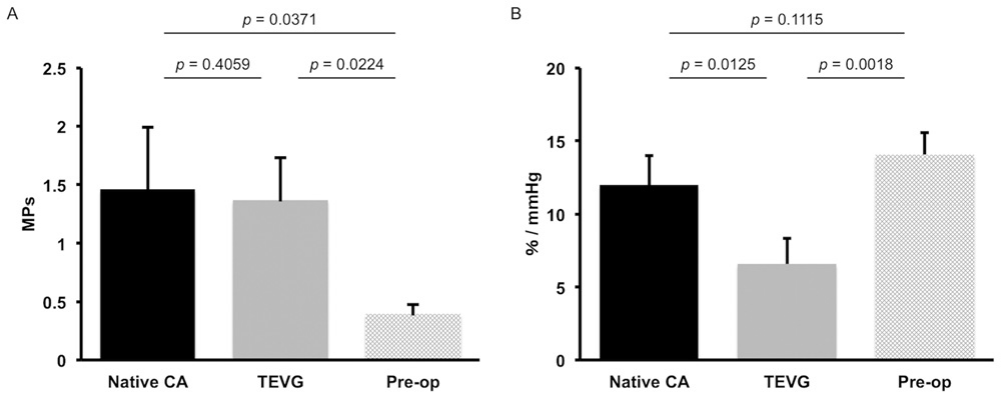
Mechanical properties. The burst pressure (A) and compliance (B) of the native CA, TEVG, and pre-operation/implantation were indicated respectively. There was no significant difference in burst pressure between the native CA and TEVG at 6 months. However, the TEVG at 6 months was significantly less compliant when compared to the native CA.

Compared the native CA, the burst pressure of the graft pre-operation was significantly less (Fig 3A, 1.46 ± 0.52MPa vs. 0.39 ± 0.08MPa, *p* = 0.0371). There was also a statistically significant difference in burst pressure between the TEVG at 6 months and graft pre-operation (Fig 3A, 1.37 ± 0.36MPa vs. 0.39 ± 0.08MPa, *p* = 0.0224). However, there was no significant difference in burst pressure between the native CA and TEVG at 6 months (Fig 3A, 1.46 ± 0.52MPa vs. 1.37 ± 0.36MPa, *p* = 0.4059). With regards to compliance, there was no significant difference between the native CA and graft pre-operation (Fig 3B, 11.98 ± 2.02% vs. 14.04 ± 1.50%, *p* = 0.1115). However, the TEVG at 6 months was significantly less compliant when compared to the native CA (Fig 3B, 6.58 ± 1.76% vs. 11.98 ± 2.02%, *p* = 0.0125) and graft pre-operation (Fig 3B, 6.58 ± 1.76% vs. 14.04 ± 1.50%, *p* = 0.0018).

#### Histological results

**Fig 4:**
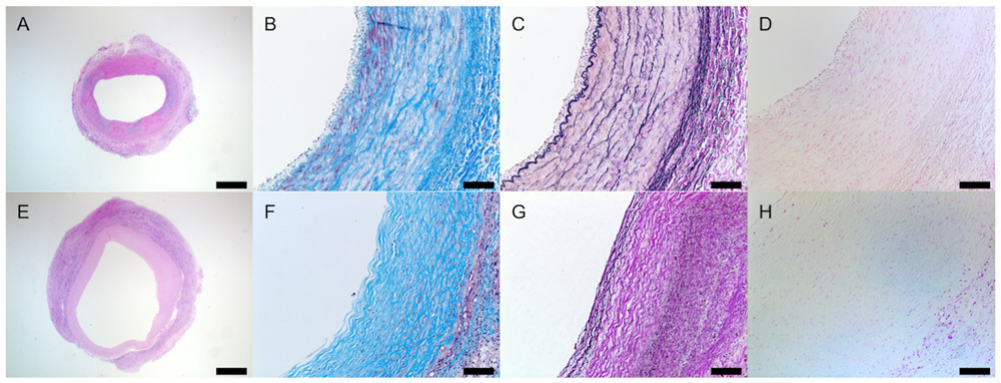
Histological assessment of vascular neotissue formation at 6 months. The vascular neotissue including collagen and elastin deposition is similar to native carotid artery (CA) without ectopic calcification. Representative pictures are shown for H&E staining (A, E), Masson’s trichrome staining (B, F), Hart’s staining (C, G), and von Kossa staining (D, H). Native CA (A-D) is compared to nanofiber PCL/CS TEVGs (E-H). The scale bar represents a length of 1,000μm for A and E, and 100μm otherwise.

**Fig 5:**
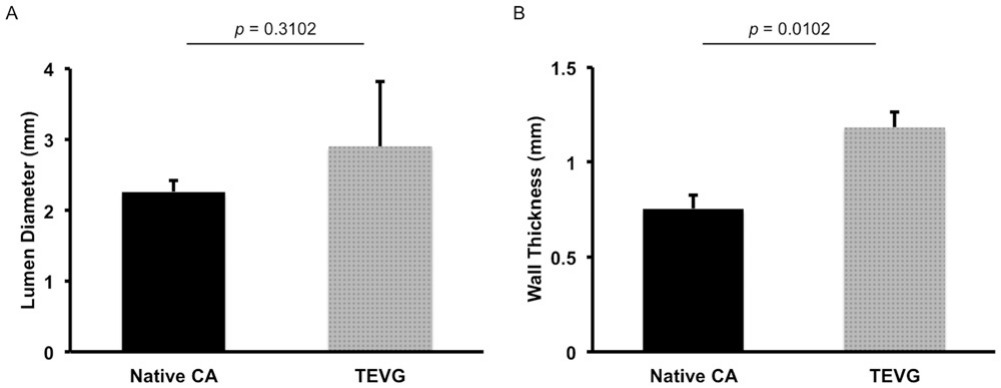
Lumen diameter and wall thickness analysis. Histomorphometric comparison of the inner diameter (A) and wall thickness (B) between the nanofiber PCL/CS TEVGs and the native CA revealed no significant difference in the inner diameter and a significant difference in the wall thickness.

**Fig 6:**
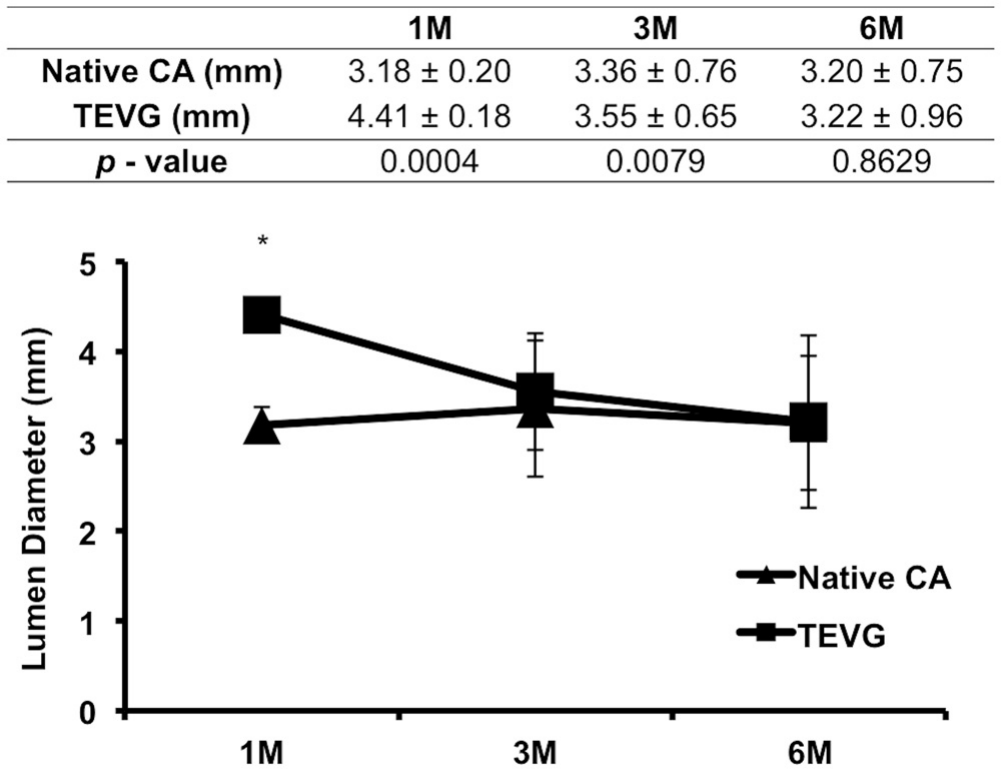
Inner diameter over time by serial ultrasound. Comparison between the TEVG and native carotid artery (CA) in patent TEVGs revealed that the TEVG lumen diameter gradually mimics the native CA. There was a statistically significant difference at 1 month, but no significant differences at 3 and 6 months after implantation. The symbol (*) denotes statistical significant differences between the native CA and TEVG at 1 month.

H&E staining showed that cellular infiltration was extensive in the TEVG and therefore the pore size/fiber diameter of the scaffold was enough to allow for cell infiltration (Fig 4A and 4E), and a patency rate of 66.7% (4/6) without any incidence of graft dilatation or rupture. The inner diameter of nanofiber TEVG and native CA measured 2.90 ± 0.92mm and 2.25 ± 0.16mm respectively (Fig 5A, *p* = 0.3102). Ultrasound measurements revealed no significant difference between the TEVG and native CA diameter at 3 and 6 months, as the TEVG gradually remodeled to mimic the CA lumen in size (Fig 6). However, there was a statistically significant difference in wall thickness between the TEVG and native CA (Fig 5B, TEVG: 1.18 ± 0.08mm vs. native CA: 0.75 ± 0.07mm, *p* = 0.0102).

**Fig 7:**
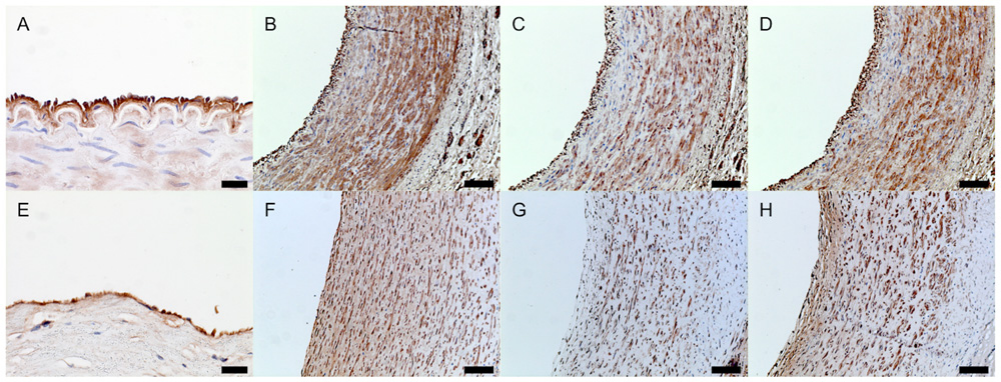
Endothelialization and smooth muscle cell (SMC) differentiation in TEVG neotissue at 6 months. TEVG ECs and SMCs were mature and well-organized, which reflected the native carotid artery (CA) in area, distribution, and density. Representative photomicrographs are shown for vWF (A, E), α- smooth muscle actin (SMA) (B, F), calponin (C, G), and myosin heavy chain (MHC) (D, H) for native CA (A-D) and nanofiber PCL/CS TEVGs (E-H). The scale bar represents a length of 20μm for A and E, and 100μm otherwise.

On the graft lumen surface, a cellular monolayer stained positively for vWF, indicating endothelial cell layer formation (Fig 7A and 7E). The luminal surfaces of patent grafts displayed no evidence of microthrombosis.

**Fig 8:**
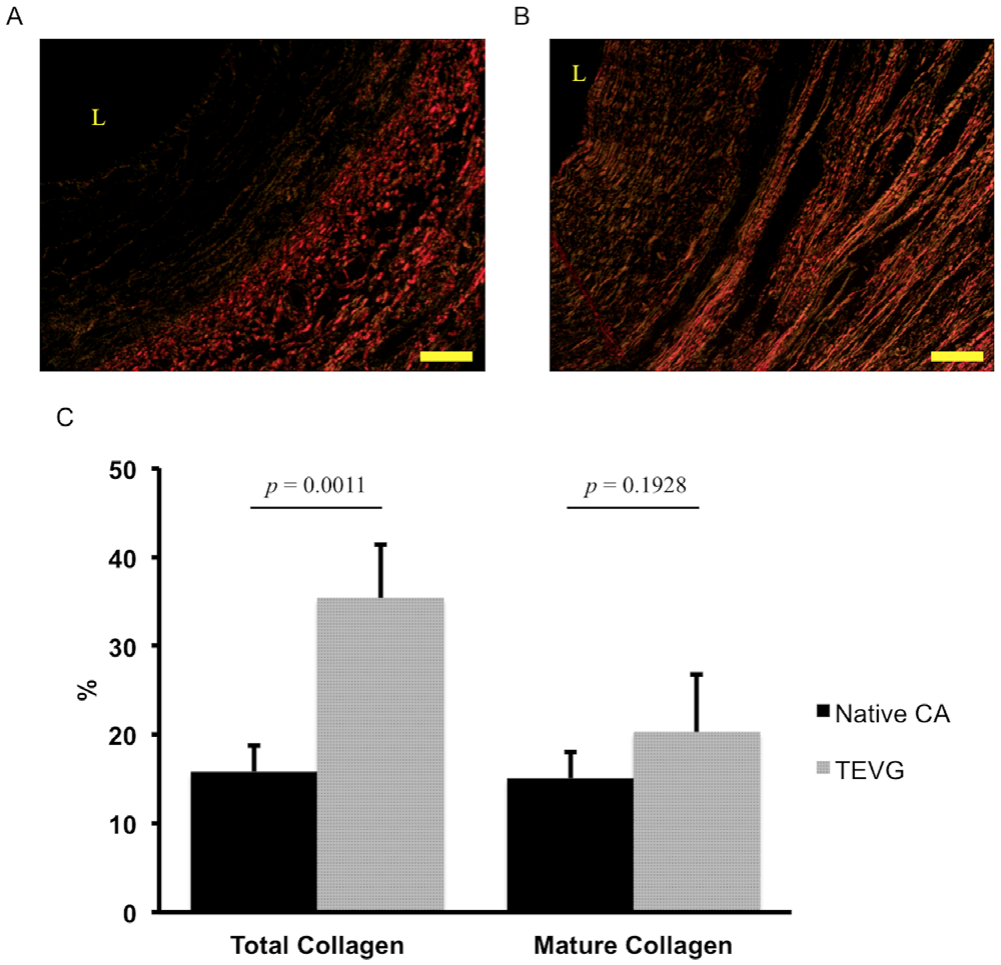
Collagen visualization and quantification. The collagen of native CA (A) and TEVG (B) were visualized by picrosirius red staining using polarized light. In collagen analysis (C), there was significantly more total collagen volume in the TEVG when compared with native CA, whereas the volume of mature collagen was similar in both the TEVG and native CA. The scale bar is represents a length of 100μm for A and B. The symbol (L) denotes the vessel lumen.

Histological assessment of extracellular matrix (ECM) constituents, including collagen and elastin, was similar to native CA. Collagen synthesis was confirmed with collagen type I and III expression. The ECM (Fig 4B and 4F), collagen deposition and density in the TEVG resembled that of native tissue. Picrosirius red staining revealed red, green, and yellow/orange fibers (Fig 8A and 8B). Red fibers were indicative of mature collagen type I fibers, whereas green fibers represented immature collagen type III fibers. Yellow/orange (intermediate between red and green) fibers characterized tissue growth and maturation representing a spectrum consisting of collagen type III (immature) to type I (mature) and provided neovessel biomechanical properties as needed as the scaffold degrades. There was significantly more total collagen volume in the TEVG when compared with native CA (Fig 8C, 35.40 ± 6.03% vs. 15.82 ± 2.92%, *p* = 0.0011), whereas the volume of mature collagen type I was similar in both the TEVG and native CA (Fig 8C, 20.30 ± 6.47% vs. 15.09 ± 2.94%, *p* = 0.1928). However, the volume of intermediary type III to type I collagen was significantly larger in the TEVG when compared with native CA (15.10 ± 7.11% vs. 0.73 ± 0.23%, *p* = 0.0068). In addition, there was no evidence of the immature collagen in both the TEVG and native CA at 6 months. Hart’s staining demonstrated that elastin was deposited within the internal and external elastic lamina of the TEVG, but it did not compare to the thickness and organization of elastin present in the native CA (Fig 4C and 4G). There was no evidence of ectopic calcification at 6 months (Fig 4D and 4H).

H&E staining observed with polarized light microscopy revealed that only 9.12 ± 5.41% of the nanofiber scaffold material remained at 6 months, and this material tended to remain between the outside of the neointima and neoadventitia.

#### Smooth muscle cell layer

We were successfully able to stain for multiple SMC phenotypes using α-SMA, calponin and MHC. Staining revealed that the organization and maturity of vascular SMCs in the TEVG were similar to that of native CA at 6 months. Contractile SMCs, identified by calponin and MHC positive staining, are characterized by elongated spindle-shaped cells. A multilayer of calponin and MHC positive cells was circumferentially organized and maintained adequate wall thickness from the subintimal to medial layers (Fig 7, native CA: C, D, TEVG: G, H). Synthetic SMCs, myofibroblasts, and many mesenchymal cell types are identified by α-SMA positive staining and show a cuboidal cobblestone-like morphology when compared to contractile SMCs. A multilayered population of α-SMA positive cells was primarily present in the neomedia, suggesting that the TEVG may not have been a completely mature neo-artery at 6 months and active vascular remodeling was still occurring at this time point (Fig 7B and 7F). Alternatively, mature SMCs could also express α-SMA, and the α-SMA-positive staining observed may be due to mature SMCs in the TEVG.

#### Macrophage infiltration

**Fig 9:**
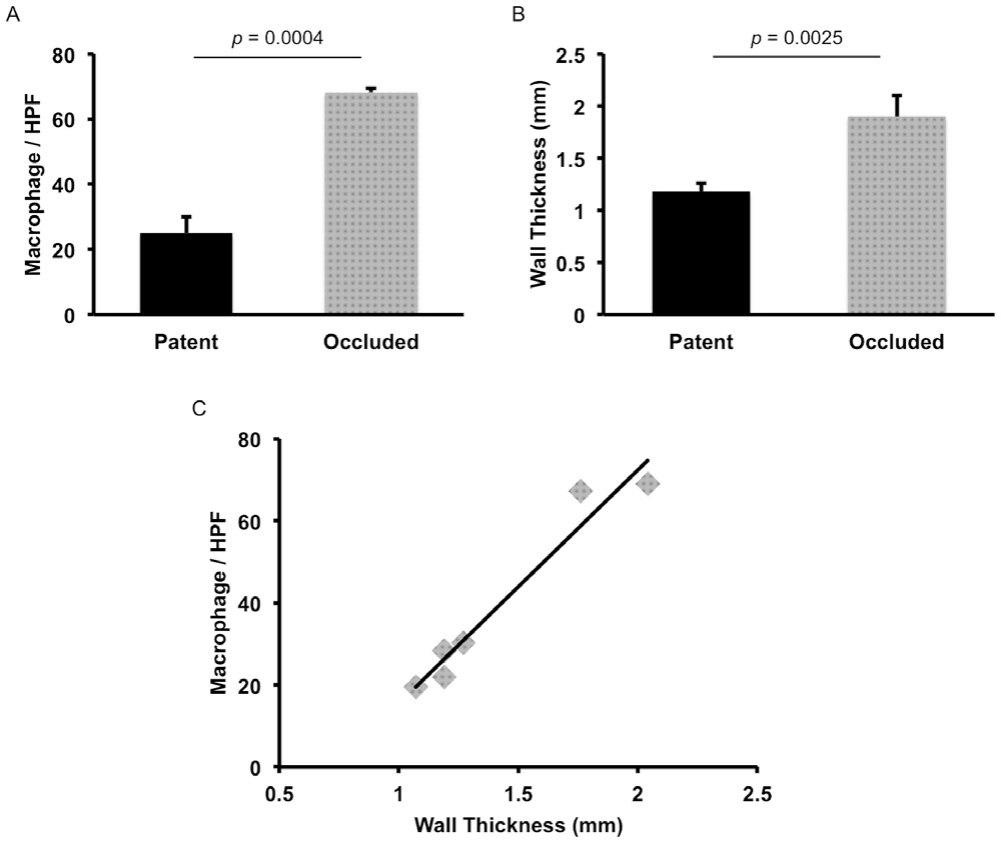
Macrophage analysis. The macrophage infiltration (A) and wall thickness (B) of patent TEVG were significantly less than that of occluded TEVGs. In addition, there was a significant positive correlation between the wall thickness of nanofiber PCL/CS TEVGs and CD68^+^ macrophages/HPF in the PCL/CS TEVGs (C).

CD68^+^ macrophages are a primary cell population in the inflammation-mediated process of vascular remodeling that occurs upon implantation of a resorbable scaffold. There was a statistically significant difference in macrophage infiltration (Fig 9A, patent group: 25.00 ± 5.08/HPF vs. occluded group: 68.13 ± 1.24/HPF, *p* = 0.0004) and wall thickness (Fig 9B, patent group: 1.18 ± 0.08mm vs. occluded group 1.90 ± 0.20mm, *p* = 0.0025) between patent and occluded TEVGs. Furthermore, we found a significant positive correlation between TEVG wall thickness and macrophage infiltration into the scaffold (Fig 9C, R^2^ = 0.9499, *p* = 0.0010).

## Discussion

The results of this study demonstrate the efficacy of a novel unseeded small-diameter arterial TEVG using electrospun PCL/CS nanofiber scaffolds in a sheep model of carotid artery interposition grafting. Serial ultrasound revealed that the initial difference between the TEVG and native CA diameters gradually resolved over the course of remodeling, and the lumen diameter of the TEVG matched that of the native CA after 3 months. The TEVG displayed an EC monolayer, contractile vascular SMCs, and ECM deposition, which all resembled the native CA. The mechanical profile of the TEVG 6 months after implantation also resembled that of the native CA. Based on these data, we suggest that the formation of well-organized vascular neotissue without aneurysm, graft rupture, or calcification at 6 months follow-up demonstrates that the TEVG underwent successful remodeling.

Electrospun PCL/CS nanofiber TEVGs have been investigated *in vitro* and *in vivo* due to their biocompatibility and favorable biomechanical properties. In 2005, an *in vitro* study investigating a hybrid PCL/CS material for tissue engineering applications demonstrated a significant improvement in mechanical properties, as well as support for cellular activity relative to PCL alone [31]. More recently, several reports have shown the effect of heparin coating or cell seeding of CS TEVGs [18, 25, 32], but there are currently no reports investigating the application of a CS TEVG *in vivo* without cell seeding or coating. Combining CS and PCL for TEVG scaffolds can provide faster degradation profiles than PCL alone due to the inherent properties of CS including its low molecular weight and its low degree of deacetylation [33]. Indeed, CS fibers are reported to have rapid *in vitro* degradation kinetics (~5 days), and degrade even faster *in vivo* [34]. Therefore, we hypothesized that the CS in our blended graft could act as a sacrificial material and would promote better cellular infiltration, vascular neotissue formation, and overcome the high density (small pore size) of traditional electrospun nanofibers made of only one polymer type. We believe that fast degradation of our CS blended scaffold led to well-organized endothelialization, SMC differentiation and proliferation, and ECM deposition. Zhou *et al*. reported that an electrospun PCL/CS nanofiber TEVG seeded with ECs demonstrated endothelialization, elastin and collagen deposition without intimal hyperplasia at 3 months in a dog model [18], thus supporting our current results.

In 1999, a porous CS-based scaffold was introduced and seeded with human coronary artery endothelial cells and smooth muscle cells. The CS scaffold demonstrated favorable cellular adhesion and promoted vascular cell proliferation [35, 36]. The possible advantages of utilizing CS for tissue engineering applications include its low cost, large-scale availability, anti-microbial properties, and biocompatibility [37]. Furthermore, CS is cationic, degrades rapidly, and shares many structural similarities to naturally occurring glycosaminoglycans (GAGs) [38]. The cationic nature of CS is hypothetically beneficial because of its ability to interact with anionic GAGs, heparin, proteoglycans, and DNA or RNA nucleotides, and other molecules. This characteristic could benefit the development of CS scaffolds with conjugated cytokines, growth factors, and other therapeutics such as transforming growth factor-beta (TGF-β) and platelet-derived growth factor (PDGF). The rapid degradation characteristic of CS are mainly attributed *in vivo* to lysozymal processing by macrophage and foreign body giant cells which leads to hydrolysis of acetylated residues and formation of non-toxic oligosaccharides, which can be incorporated in metabolic pathways or excreted [39, 40]. Moreover, the degradation rate of CS is highly dependent on its molecular weight and degree of deacetylation, parameters which could be manipulated to create an optimized tissue engineering scaffold with a rationally designed degradation profile [40, 41].

Our *in vitro* and *in vivo* results demonstrated the fast degradation and adequate mechanical properties of electrospun PCL/CS nanofiber TEVGs. The *in vitro* degradation results demonstrated a 50% decrease in ultimate tensile strength (UTS) within 2 weeks. In contrast, PCL by itself remains stable for many months *in vitro* [42]. The lack of mass loss suggests that rather than the scaffold degrading, chitosan aids the rapid loss of mechanical integrity by creating breaks in the PCL nanofibers. In addition to displaying adequate mechanical strength at 6 months, the TEVG burst pressure was comparable to that of native CA, and the compliance was about half of native CA. TEVGs abundant with ECM and collagen may lead to burst pressures comparable to that of native CA. However, TEVGs with less elastin and SMC volumes than that of native CA may lead to low compliance. Nonetheless, PCL/CS TEVG compliance may closely match that of native CA once long-term remodeling is completed.

Furthermore, the TEVGs in the current study displayed very small amounts of remaining scaffold material (remaining area average 9.12 ± 5.41%) and no calcification. In contrast to our result, plain PCL scaffolds in a rat aortic interposition model developed chronic vascular calcification [19]. Vascular SMCs express transcription factors of both osteogenesis and osteoclastogenesis, can undergo osteogenic differentiation and calcification, and potentially prevent calcium deposition [43, 44]. Our previous study demonstrated that when compared to small-pore (0.7μm) nanofiber TEVGs, large-pore (30μm) TEVGs had abundantly more SMCs, displayed well-organized neointima, and better prevented calcific deposition [13]. Our results indicate that blended PCL/CS nanofiber TEVGs sufficiently formed vascular neotissue without calcification, but there is still a need for longer-term studies to evaluate these scaffolds until complete polymer degradation before clinical application can be advocated.

Several studies have investigated the patency rates of small diameter TEVGs implanted as CA conduits in large animal models [16–18, 45, 46]. We demonstrated that our blended PCL/CS (20:1 weight ratio) nanofiber TEVG was patent in 4 out of 6 cases (66.7%) using a postoperative course of aspirin. Another group with identical implantation and postoperative care methods investigated a PCL/CS nanofiber (1:1 weight ratio) TEVG with EC seeding. 5 out of 6 cases (83.3%) were patent at 3 months, however only 1 out of 6 control TEVGs without outgrowth EC seeding were patent, and the occluded cases were due to thrombosis within 2 months [18]. We suggest that these outcomes are due to the cationic nature of CS that leads to poor hemocompatibility due to undesirable adhesion with anionic platelets [25]. Therefore, the PCL to CS ratio becomes a more important factor for unseeded TEVG applications *in vivo* because the immediate risk of thrombosis must be weighed against the degradation rate and scaffold pore size (among other variables) to create the optimal cell-free construct [18, 25, 31].

With regards to long-term vascular remodeling, we found a significant positive correlation between TEVG wall thickness and CD68^+^ macrophage infiltration into the scaffold (R^2^ = 0.95, *p* = 0.0010). Our previous studies suggested that excessive macrophage infiltration contributes to vascular neotissue hyperplasia, which leads to stenosis and graft occlusion [47, 48]. However, acute thrombosis cannot be ruled out in this study as a cause of graft occlusion because ultrasound revealed that both stenotic TEVGs were occluded by 1-month follow-up. It is difficult to ascertain whether the cause of stenosis/occlusion in our 6-month study is due to acute thrombosis, neointimal hyperplasia, or a combination of both processes. Even though cell seeding improves TEVG patency, including such a process diminishes a scaffold’s “off-the-shelf” potential and greatly increases the regulatory burden required to bring the product to market.

Vascular SMCs can perform both contractile and synthetic functions [49], and thus have the necessary flexibility to efficiently assume a spectrum of phenotypes under different physiological and pathological conditions. SMCs are characterized by changes in morphology, proliferation and migration rates, and the expression of different marker proteins [49, 50]. Immunohistochemistry of the PCL/CS scaffold encouragingly demonstrated the completion of SMC differentiation and migration *in vivo* at 6 months. In the TEVG, a circumferentially aligned multilayer of contractile SMCs was similar in thickness and density to that found in the native CA. However upon searching the literature, there were no representative staining images of vascular SMC maturation in biodegradable vascular scaffolds in a sheep model. For the first time, we show that CS’s fast degrading properties encourages transanastomotic [51] or transmural [52] migration of synthetic SMCs and their differentiation and maturation in a sheep CA model.

## Limitations

While the results of this study are promising and clearly encourage further investigation, this experiment was limited by the short length of the scaffolds and small number of animals in the study. Large animal studies investigating small-diameter TEVG longer than 5cm in length indicate low patency at the intermediate follow-up time point [18, 53]. Furthermore, though patency rates are better during short-term follow-up, it is difficult to evaluate neotissue formation due to the amount of scaffold remaining at these time points. We used short length TEVGs (1.5cm) in a large animal model to maintain graft patency and explanted them at 6 months to evaluate vascular neotissue formation. Future studies should investigate the degree of neotissue formation at extended follow-up time points after complete scaffold degradation. In addition, cell-free TEVGs longer than 5cm should be implanted and evaluated for graft patency and remodeling before initiation of human clinical trials. Despite our study limitations, the cell-free electrospun PCL/CS nanofiber TEVGs investigated in this report provided important information regarding vascular remodeling, occlusive complications and the degree of neotissue formation at the 6-month time point.

## Conclusions

Unseeded electrospun blended PCL/CS nanofiber TEVGs might be an attractive alternative to small diameter prosthetic vascular grafts for the management of coronary artery and peripheral vascular disease. The fast degradation profile leads to rapid cellular infiltration, improved vascular remodeling, and neotissue formation without calcification or aneurysm. We are the first to demonstrate vascular SMC maturation in an electrospun biodegradable scaffold in the sheep model. Despite study limitations, such as graft length and time course, our novel hybrid PCL/CS TEVG warrants further investigation due to its great clinical potential and “off-the-shelf” availability.
